# Opportunistic infections and immune reconstitution inflammatory syndrome (IRIS) in HIV infected patient – late presenter in cART era: case report

**DOI:** 10.1186/1471-2334-14-S4-P43

**Published:** 2014-05-29

**Authors:** Corina Mitroi-Maxim, Elena Dumea, Stela Halichidis, Sorin Rugină, Elisabeta Otilia Benea, Ruxandra Moroti, Eugenia Muja, Ghiulendan Resul, Elena Dantes, Simona Claudia Cambrea

**Affiliations:** 1 Clinical Hospital of Infectious Diseases, Constanța, Romania; 2 Ovidius University, Constanța, Romania; 3 National Institute for Infectious Diseases “Prof. Dr. Matei Balş”, Bucharest, Romania; 4 Clinical Pneumology Hospital, Constanța, Romania

## 

HIV-infected individuals are at high risk of developing numerous opportunistic infections. The severity of these infections may increase proportional to the immunosuppression degree. We must pay special attention to immune reconstitution inflammatory syndrome (IRIS) in order to prevent worsening symptoms and death. HIV coinfection is associated with high mortality rate despite effective antiretroviral therapy.

We present the case of a 42 male patient who was diagnosed with AIDS and pulmonary tuberculosis in 2011 in our clinic. Our theme includes clinical, biological, immunological, virological evolution and therapeutics of this patient.

He was a late-presenter patient with advanced immunodepression at baseline: low CD4 count, increased viral load in blood and cerebrospinal fluid. After a month of tuberculosis treatment, antiretroviral therapy was instituted according to guidelines. During one year the patient subsequently developed IRIS and, one by one, several opportunistic infections, including CNS involvement.

Thus he presented: *Cryptococcus neoformans* meningoencephalitis resistant to fluconazole with multiple relapses, TB meningoencephalitis, severe form of CMV disease with encephalitis, demyelinating lesions, necrotic ulcerative stomatitis and esophagitis with HSV, systemic candidiasis, severe bacterial infections with multidrug-resistant germs. Diagnoses were based on the usual investigations, including molecular biology techniques (RT-PCR: *Mycobacterium tuberculosis*, JC virus, *Cryptococcus neoformans*), viral resistance testing, PLEX-ID, MRI. Viral PLEX-ID identified the presence of Epstein Barr virus in CSF at a high level. Opportunistic infections occurred imposed specific therapy and reconsideration of antiretroviral therapy with CNS penetration ARV (score Letendre). The patient was adherent to ARV therapy. The evolution was initially favorable under specific therapy with clinical, immunological and virological improvement. Unfortunately, about 10 months after diagnosis, the patient developed CNS lymphoma possibly in relationship with increased levels of Epstein Barr virus in CSF, having fatal outcome.

The evolution of this case pointed out once again that in a patient with AIDS at the time of initiating ART, it should be considered the possibility of IRIS and future opportunistic infections, associated with a poor prognosis. Therefore it is important to detect persons with HIV infection in the early stages of the disease in order to obtain a favorable evolution.

